# Near-Isotropic Local Attosecond Charge Transfer within the Anisotropic Puckered Layers of Black Phosphorus

**DOI:** 10.1021/acs.jpclett.3c01977

**Published:** 2023-09-22

**Authors:** Robert Haverkamp, Stefan Neppl, Alexander Föhlisch

**Affiliations:** † Institute for Methods and Instrumentation for Synchrotron Radiation Research, Helmholtz-Zentrum Berlin für Materialien und Energie GmbH, Albert-Einstein-Straße 15, Berlin 12489, Germany; ‡ Institute of Physics and Astronomy, University of Potsdam, Karl-Liebknecht-Straße 24/25, Potsdam 14476, Germany

## Abstract

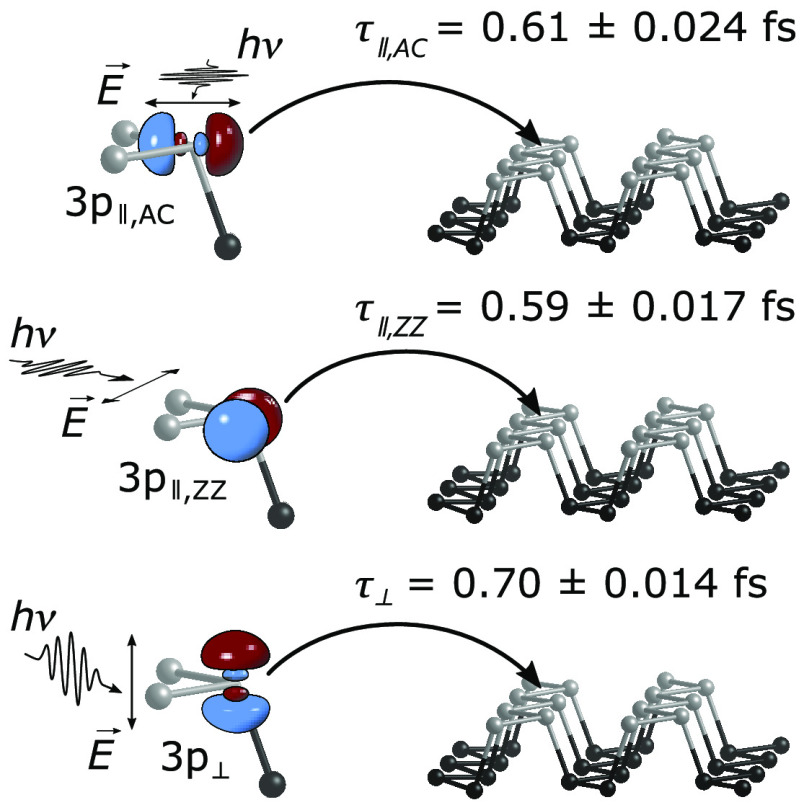

Black phosphorus possesses useful two-dimensional (2D) characteristics of van der Waals coupled materials but additionally features an in-plane anisotropic puckered layer structure that deviates from common 2D materials. Three distinct directions exist within the lattice of black phosphorus: the in-plane armchair and zigzag directions and the out-of-plane direction, with each distinct phosphorus 3p partial density of states. This structural anisotropy is imprinted onto various collective long-range properties, while the extent to which local electronic processes are governed by this directionality is unclear. At the P L_1_ edge, the directional selectivity of the core-hole clock method was used to probe the local charge transfer dynamics of electrons excited into the 3p-derived conduction band on an attosecond time scale. Here we show that the surprisingly small anisotropy of 3p electron transfer times reflects the similarly small differences in the 3p-derived unoccupied density of states caused by the underlying phosphorus bonding angles within the puckered layers.

Unlike the perfectly planar conformation prevailing in sp^2^-bonded two-dimensional (2D) materials, sp^3^-hybridized 2D materials such as black phosphorus (BP) possess a naturally puckered crystal structure. Under ambient conditions, BP is the thermodynamically most stable allotrope of the group V element phosphorus (P), which has emerged as a promising material for electrochemical energy storage,^1,2^ electronic,^3,4^ and photonic applications.^5,6^ Bulk BP is a layered material whose layer structure consists of P atoms arranged in a puckered hexagonal lattice^7^ as illustrated in Figure 1a. While the van der Waals (vdW) interlayer interaction is comparably weak, with a layer-to-layer spacing (*b_1_*) of 5.46 Å,^8^ within each layer, the P atoms covalently bond to three adjacent P atoms through sp^3^ hybridization involving the 3s and 3p orbitals.^9^ The geometry of a single layer of BP in top and lateral views is illustrated in panels b and c, respectively, of Figure 1, where the lengths of the P–P bonds are 2.21 Å (*a*_*1*_) and 2.16 Å (*a*_*2*_) and the corresponding bond angles are 98.2° (θ*_1_*) and 103.7° (θ_*2*_),^8^ respectively.

**Figure 1:**
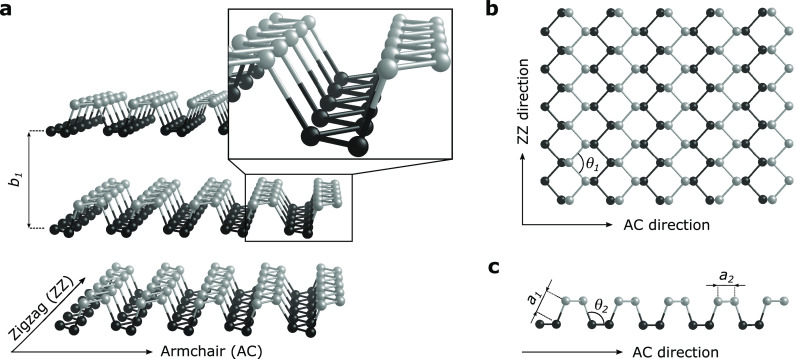
Crystallographic structure of black phosphorus (BP). (a) Perspective view of the puckered hexagonal lattice structure of BP with its two distinct in-plane directions: armchair (AC) and zigzag (ZZ). (b) Top view and (c) lateral view of single-layer BP. Phosphorus atoms in the upper plane of a layer are denoted by a lighter gray compared to those in the lower plane. Structural parameters are defined in the text.

The layered arrangement of corrugated P atoms leads to structural anisotropy not only between the in-plane and out-of-plane direction but also between the two inequivalent high-symmetry in-plane directions: armchair (AC) and zigzag (ZZ) (inset of Figure 1a). The in-plane AC and ZZ directions are oriented at a right angle to each other. The stacked crystal structure of semiconducting BP gives rise to a layer-dependent band gap ranging from ∼0.3 eV (bulk) to ∼1.7 eV (monolayer).^10^ Considering the orbital components, the 3p-derived band states dominate the electronic band structure with significant weight both below and above the Fermi level (*E*_*F*_).^11^ Below *E*_*F*_, a distinct anisotropic dispersion of 3p valence bonding states exists, especially between the out-of-plane and in-plane directions.^11−13^ Above *E*_*F*_, the constituent 3p orbitals forming the conduction band also exhibit a directional dependence, which is, however, much less pronounced.^11−13^

The structural anisotropy is a central aspect of BP and is also reflected to varying degrees in its inherent properties. So far, directional dependence has been reported for the optical response (absorption,^11,14^ transmission,^11^ and reflection^11,15^) of BP as well as for various collective long-range properties on the macroscopic length scale like the mechanical (fracture stress^16^ and Young’s modulus^16,17^) and vibrational characteristics,^18,19^ the thermal properties (expansion^20^ and conductivity^21,22^), and the electrical conductance^16,23^ and the charge mobility.^14,23^

With regard to the highly anisotropic crystal structure and the variably pronounced anisotropy in the band structure, the question of the extent to which ultrafast charge transfer (CT) within the puckered layers of BP already exhibits directional dependence at the atomic level arises. Here we address this question by selectively probing the local CT dynamics in oriented, single-crystal, bulk BP along all three crystal directions: the out-of-plane (⊥) and the in-plane armchair (∥_AC_) and zigzag (∥_ZZ_) direction. Photoinduced CT was measured using the core-hole clock (CHC)^24,25^ approach at the P L_1_ absorption edge. The viability of the CHC method to reveal CT dynamics on a sub-femtosecond time scale with orbital specificity has recently been demonstrated on a variety of other 2D layered materials.^26−29^ Despite the macroscopic structural anisotropy of black phosphorus, only a weak local ultrafast charge transfer anisotropy is found, which results from the atomic arrangement and bonding angles of phosphorus atoms within the puckered layered structure. In addition, this finding is corroborated by agreement with the unoccupied spatially resolved density of states in black phosphorus.

CT times are derived from resonantly excited P 2s core electrons into P 3p orbitals by using the known P 2s core-hole lifetime as a reference clock for the decay processes of the transient core-excited state. Decay events before and after the occurrence of CT of the resonantly excited electron show different characteristic dispersive behavior. The kinetic energy of the ejected electron depends linearly on the photon energy if the excited P 3p electron remains in an atomically localized state during the decay (Raman channel). A constant kinetic energy is observed if the excited P 3p electron is delocalized within the conduction band during the core-hole decay process, i.e., if CT occurred (Auger channel). The principle of the CHC method on BP is schematically presented in Supplementary Figure 1. Thus, scanning the photon energy across the P L_1_ X-ray absorption edge allows one to unambiguously separate and quantify the different decay channels. The intensity ratio of the localized Raman (*I*_*Raman*_) and the delocalized Auger (*I_Auger_*) channel in the deexcitation spectrum in combination with the P 2s core-hole lifetime τ*_P 2s_* of 0.65 ± 0.065 fs^30^ yields the charge transfer time τ*_CT_* according to the CHC rate model τ*_CT_* = *I*_*Raman*_/*I*_*Auger*_ · τ*_P 2s_*.^24^

Resonant core excitation via the P 2s → P 3p dipole-allowed transition enables orbital selectivity by the use of linearly polarized X-rays. With the X-ray electric field vector (*E⃗*) aligned parallel or perpendicular to the sample surface, either the in-plane P 3p_∥_ or out-of-plane P 3p_⊥_ conduction band states are populated. From low-energy electron diffraction measurements (Supplementary Figure 2), the two in-plane crystal orientations can be identified. Therefore, by rotating the sample 90° with respect to the X-ray polarization under otherwise unchanged conditions, we can further selectively measure the CT times of electrons excited into in-plane P 3p_∥_ orbitals pointing along the ZZ direction (P 3p_∥,ZZ_) or the AC direction (P 3p_∥,AC_).

Figure 2 depicts schematically how linearly polarized X-rays prepare P 3p_⊥_, P 3p_∥,ZZ_, and P 3p_∥,AC_ orbital population together with the corresponding P L_1_L_2,3_M_1,2,3_ Coster–Kronig (CK) autoionization spectra obtained for resonant core-to-bound excitation as a function of photon energy. Figure 2 shows representative data sets. The consistency and robustness of our results are demonstrated by additional measurements, presented in Supplementary Figure 3. The photon energy was tuned across the P L_1_ absorption edge from 182 eV to 200 eV, and the CK autoionization spectra are monitored from 28 eV to 58 eV kinetic energy (*E*_*kin*_). The P L_1_ X-ray absorption spectrum is shown in Supplementary Figure 4, with the resonance maximum at 191.2 ± 0.1 eV. Considering the P 2s binding energy of 188.5 eV (Supporting Information), this corresponds to an energetic position of the resonantly excited P 2s electron of 2.6 ± 0.1 eV above *E*_*F*_. To decompose and quantitatively analyze the branching of the CK autoionization decay channels into the Raman- and Auger contributions, we performed a curve-fitting procedure^24,25^ as described in the Supporting Information (Supplementary Figure 5 and Supplementary Figure 6).

**Figure 2:**
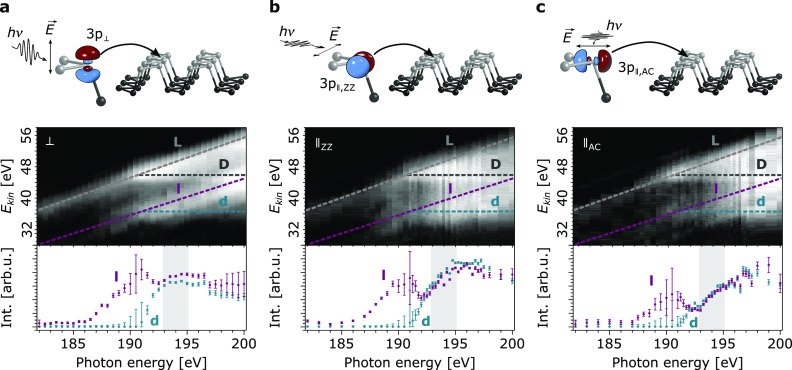
Directional preparation of (a) P 3p_⊥_, (b) P 3p_∥,ZZ_, and (c) P 3p_∥,AC_ excited states in BP, by means of linearly polarized X-rays, and the corresponding P L_1_L_2,3_M_1,2,3_ CK autoionization spectra as a function of photon energy. The Raman channels l (P 2p^–1^3s^–1^3p^1^) and L (P 2p^–1^3p^–1^3p^1^) as well as Auger channels d (P 2p^–1^3s^–1^deloc^1^) and D (P 2p^–1^3p^–1^deloc^1^) are indicated. The branching ratios of the respective l- and d-channels are plotted below each autoionization spectrum. Error bars represent the spectral fit uncertainty. The relevant photon energy region from 193 eV to 195 eV, in which CT times are reliably extractable, is highlighted. The spectral contributions of the direct P 2p photoionization have been subtracted.

In the CK autoionization spectra, the localized pre-CT Raman channels P 2p^–1^3s^–1^3p^1^ (l) and P 2p^–1^3p^–1^3p^1^ (L) as well as the delocalized post-CT Auger channels P 2p^–1^3s^–1^deloc^1^ (d) and P 2p^–1^3p^–1^deloc^1^ (D) are indicated. For photon energies above the P L_1_ edge maximum, the d- and D-channels emerge at kinetic energies of 36.2 eV (d) and 45.2 eV (D), respectively. The intensities of the respective l- and d-channels as a function of photon energy are plotted in the bottom panels in Figure 2. The quantitative evaluation of the directional CT times is exclusively based on these channels, as they are the spectrally pure autoionization final states.^24^

In the top panel of Figure 3, the relevant section of the derived CT times in out-of-plane direction τ_*⊥*_ (Figure 3a) as well as two in-plane directions τ_*∥,ZZ*_ (Figure 3b) and τ*_∥,AC_* (Figure 3c) are depicted. The CT times over the entire analyzed photon energy range are shown in Supplementary Figure 7. The photon energy range from 193 eV to 195 eV, i.e., just above the P L_1_ absorption edge maximum for resonant excitation into the conduction band minimum, is used to calculate a weighted average value for τ*_CT_* in each direction and is highlighted in Figures 2 and 3.

**Figure 3:**
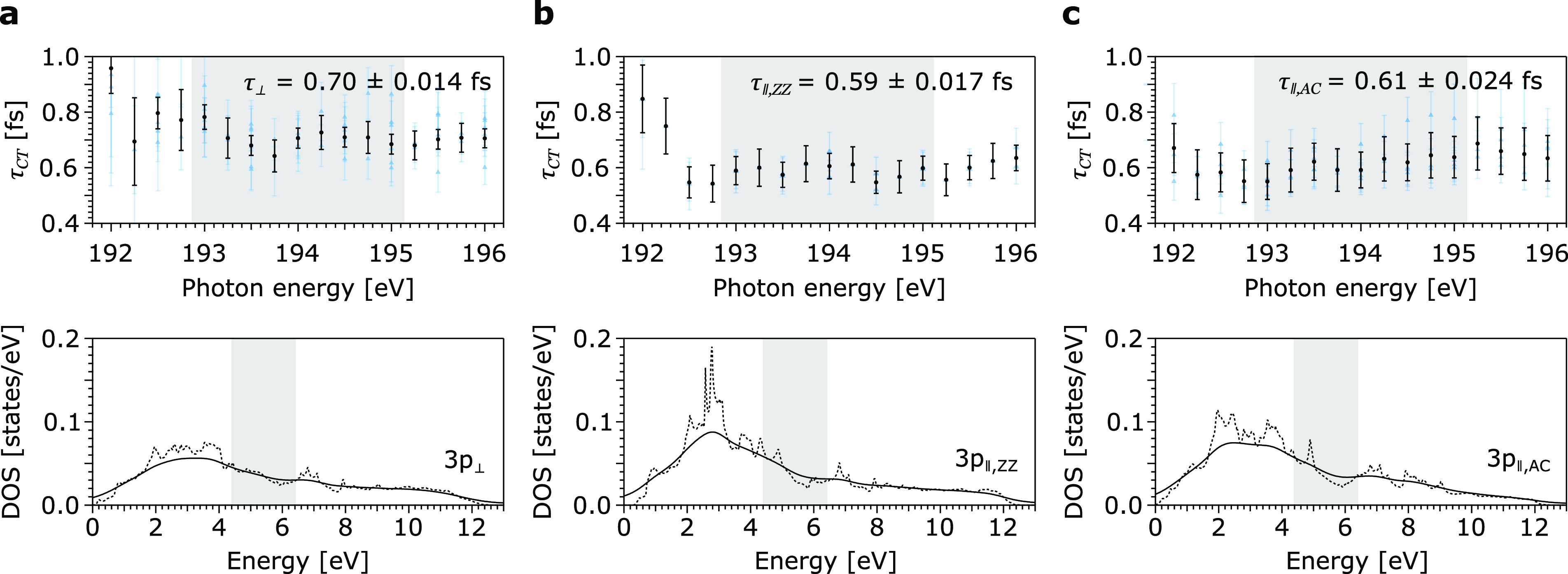
Photon energy-dependent CT times in BP in the (a) out-of-plane τ_*⊥*_ and (b) in-plane zigzag τ*_∥,ZZ_* and (c) armchair τ*_∥,AC_* directions, with the calculated conduction band DOS for P 3p_⊥_, P 3p_∥,ZZ_, and P 3p_∥,AC_ orbitals in the bottom panel. Extracted τ*_CT_* values obtained from individual P L_1_L_2,3_M_1,2,3_ spectra (blue triangles) were used to calculate weighted average values (black circles). Error bars for individual measurements result from Gaussian error propagation through the CHC analysis. Error bars for mean values were calculated from the standard deviation of the weighted mean. The data from the DOS calculations were taken from ref (12) (*E*_*F*_ = 0 eV). The solid line is obtained by a Lorentzian convolution (1.01 eV FWHM) with the calculated DOS (dashed line) to account for the 2s lifetime broadening.^30^ The relevant photon energy region from 193 eV to 195 eV, respectively, from 4.4 eV to 6.4 eV above *E*_*F*_, is highlighted.

This systematic study reveals charge transfer times of 0.70 ± 0.014 fs (τ*_⊥_*) in the out-of-plane direction and in-plane charge transfer times of 0.59 ± 0.017 fs in the ZZ direction (τ*_∥,ZZ_*) and 0.61 ± 0.024 fs in the AC direction (τ*_∥,AC_*). It is found that (i) the CT from out-of-plane P 3p_⊥_ orbitals is decelerated by ∼18.6% compared to τ*_∥,ZZ_* and (ii) the CT from in-plane P 3p orbitals has no distinct anisotropy within the error of the measurement. Overall, we observe that the local CT emanating from the resonantly core-excited P 2s state proceeds on a sub-femtosecond time scale without a pronounced directional dependency.

For the interpretation of our findings, we note that CT times measured with the CHC approach reflect the degree of electron delocalization in the core-excited state. This depends on both the spatial and energetic character of the orbital into which the core electron is initially excited and its coupling to the conducting state, to which it is eventually transferred. The CHC approach therefore provides a quantitative measure of the interatomic electronic coupling strength in the excited state of the system. Because the puckered hexagonal structure of BP consists of only P atoms, this interatomic coupling is determined by a lattice consisting exclusively of chemically equivalent directional P–P bonds, formed predominantly by P 3p states.

Considering the vdW layered structure with the two inequivalent high-symmetry in-plane directions, a higher degree of anisotropy might be expected to be reflected in the interatomic coupling and, thus, the CT times. However, this macroscopically depicted structural anisotropy of BP is not present on the atomic level, i.e., in the local bonding configuration of the P atoms (top panel in Figure 2). In fact, the microscopic bonding environment of the P atoms within the BP layer is suitable to account for the observed near-isotropic CT times.

From a geometrical point of view, upon in-plane excitation, irrespective of the localization of the in-plane 3p_∥_ orbital into which the 2s core electron is excited, the P 3p_∥,ZZ_ and P 3p_∥,AC_ orbitals have similar projections onto equivalent in-plane covalent P–P bonds due to the nearly rectangular bond angle 96.3° (θ*_1_*). Consequently, similar delocalization pathways for excitation along the ZZ and AC directions are probed, and the measured CT times in both in-plane directions (τ*_∥,ZZ_* and τ*_∥,AC_*) should not show any distinct directional dependence. This microscopic character of the 3p orbital bonding is also suitable to explain the observed out-of-plane τ*_⊥_* deceleration. Due to the increased interatomic distance *a_1_*, the spatial overlap of 3p orbitals of neighboring P atoms decreases, which is reflected in a lower probability for CT to occur, resulting in a less efficient out-of-plane CT.

Recently, a similar interpretation has been proposed to explain hard X-ray CHC-derived CT times for the vdW-coupled transition metal dichalcogenide MoS_2_.^31^ By systematically measuring the layer dependence of the directional CT, it is found that the interlayer vdW interaction is negligible for electrons excited to S 3p_⊥_ states and that the femtosecond electron delocalization pathway in the in-plane and out-of-plane directions is due to the intralayer Mo–S covalent bonds formed by S 3p and Mo 4d states. In contrast to MoS_2_, the electron delocalization pathway in BP through the 3p orbital bonding is even more clearly defined because the crystal lattice consists exclusively of chemically equivalent P–P bonds.

Further, the microscopically nearly isotropic 3p bonding characteristics are also reflected in the weak anisotropic 3p-dominated conduction band structure of BP. The orbital projections of the density of states (DOS) for P 3p_⊥_, P 3p_∥,ZZ_, and P 3p_∥,AC_ states in bulk BP above the *E*_*F*_, obtained from theoretical calculations,^12^ are shown in the bottom panel of Figure 3 (dashed line). To model the propagation of the resonantly excited 2s core electron within the unoccupied 3p DOS, the bulk BP conduction band states were convoluted with the 2s lifetime broadening (solid line), represented by a Lorentzian profile with a 1.01 eV full width at half-maximum (FWHM),^30^ which accounts for the initial excitation process. The highlighted energy region from 4.4 eV to 6.4 eV above *E*_*F*_ corresponds to the photon energy range of 193 eV to 195 eV (derived from the energy position of the P L_1_ X-ray absorption maximum and the P 2s core level, shown in the Supporting Information) and marks the range in which the CHC spectral signatures are reliably quantifiable in our experiment. The Lorentzian convoluted DOS in this region was integrated to allow a quantitative analysis and comparison with the experimentally obtained CT rates. Compared to the P 3p_∥,ZZ_ states, the following ratios are obtained: (i) the amount of P 3p_⊥_ states is decreased by ∼13.2%, and (ii) the in-plane 3p DOS barely shows any directionality with only ∼0.8% more P 3p_∥,AC_ states. These DOS ratios closely agree with the experimental τ*_CT_* ratios of P 3p electrons and reflect the probability of CT to occur, i.e., the larger the empty DOS, the higher the CT rates.^32,33^ Minor deviations of the theoretical DOS ratios compared to the experimental τ*_CT_* ratios might be caused by modifications in the local electronic structure due to the presence of the P 2s core vacancy.

The agreement between the experimentally obtained CT rates and the theoretically predicted DOS ratios^12^ corroborates our findings and interpretation. It is demonstrated that for BP with a well-defined delocalization pathway, within the puckered layers near-isotropic local CT at the atomic level prevails, caused by the local 3p orbital overlap, i.e., the local electronic structure at the excited atom site. Our results highlight the disparity between the macroscopic and microscopic crystal structures. They suggest that weakly anisotropic to near-isotropic properties might originate from the microscopic crystal structure, i.e., the local P–P bond arrangement and/or the conduction band structure. Highly anisotropic properties might rather originate from the collective long-range interactions within the macroscopic crystal lattice and/or the distinctly anisotropic valence band structure. As an elemental sp^3^-hybridized material with in-plane anisotropy, BP might serve as a prototypical system with relevance to analogous 2D materials with a naturally puckered crystal structure.

## Methods

*XPS Measurements*. All experimental data presented in this work have been acquired at the “FEMTOSPEX Molecules and Surfaces” endstation,^34^ installed at the BESSY II soft X-ray UE56/1 PGM beamline during the single-bunch operation mode. The beamline provides adjustable X-ray polarizations (linear, vertical, and horizontal as well as circular) with a focus spot size on the sample of approximately 200 μm × 200 μm. Absolute photon energy calibration was obtained by an argon gas cell installed in the beamline. The data have been collected under ambient-temperature and ultra-high-vacuum (UHV) conditions at a base pressure in the measurement chamber of 2 × 10^–10^ mbar throughout the whole measurement time. The presented data have been measured under a grazing X-ray incidence angle of 15°. A VG Scienta-Omicron angle-resolved time-of-flight (ARTOF) electron spectrometer with a 60° acceptance angle lens system was used.^35^ In addition, preliminary measurements have been conducted at the LowDosePES endstation at BESSY II dipole beamline PM4.^36^

*Sample Preparation*. Two commercially available (HQ Graphene and Smart Elements) high-purity (>99.995%) bulk BP single-crystal samples were prepared by mechanically exfoliating the crystal under UHV conditions at a pressure of ∼2 × 10^–10^ mbar to ensure a pristine surface. We confirm the sample quality, its structural integrity, and the absence of contamination by means of *in situ* XPS measurements of the valence band, P 2p and P 2s core levels, and survey scans. Further details are given in the Supporting Information (Supplementary Figure 8).

## Data Availability

The data related to this letter are available from the corresponding authors upon request.

## Supporting Information Available

Experimental details, methods, and additional experimental results that are not included in the main text (PDF)
