# Supplementary material for: Near-Isotropic Local Attosecond Charge Transfer within the Anisotropic Puckered Layers of Black Phosphorus
Source: J Phys Chem Lett. 2023 Sep 22;14(39):8765–70. doi: 10.1021/acs.jpclett.3c01977 (PMC10561272; doi:10.1021/acs.jpclett.3c01977)
Supplement: Supplementary file 1 — jz3c01977_si_001.pdf [file jz3c01977_si_001.pdf]

Supporting Information:

Near-Isotropic Local Attosecond Charge Transfer Within the Anisotropic Puckered Layers of Black Phosphorus

Robert Haverkamp,[*,†,‡] Stefan Neppl,[*,†,‡,¶] and Alexander Föhlisch[†,‡]

†*Institute for Methods and Instrumentation for Synchrotron Radiation Research, Helmholtz-Zentrum Berlin für Materialien und Energie GmbH, Albert-Einstein-Straße 15, 12489 Berlin, Germany*

‡*Institute of Physics and Astronomy, University of Potsdam, Karl-Liebknecht-Straße 24/25, 14476 Potsdam, Germay*

¶*Current address: Laboratory for Non-Linear Optics, Paul Scherrer Institut, Forschungsstrasse 111, 5232 Villigen, Switzerland*

E-mail: Robert.Haverkamp@Helmholtz-Berlin.de; Stefan.Neppl@PSI.ch

Schematic representation of the core-hole clock principle on BP

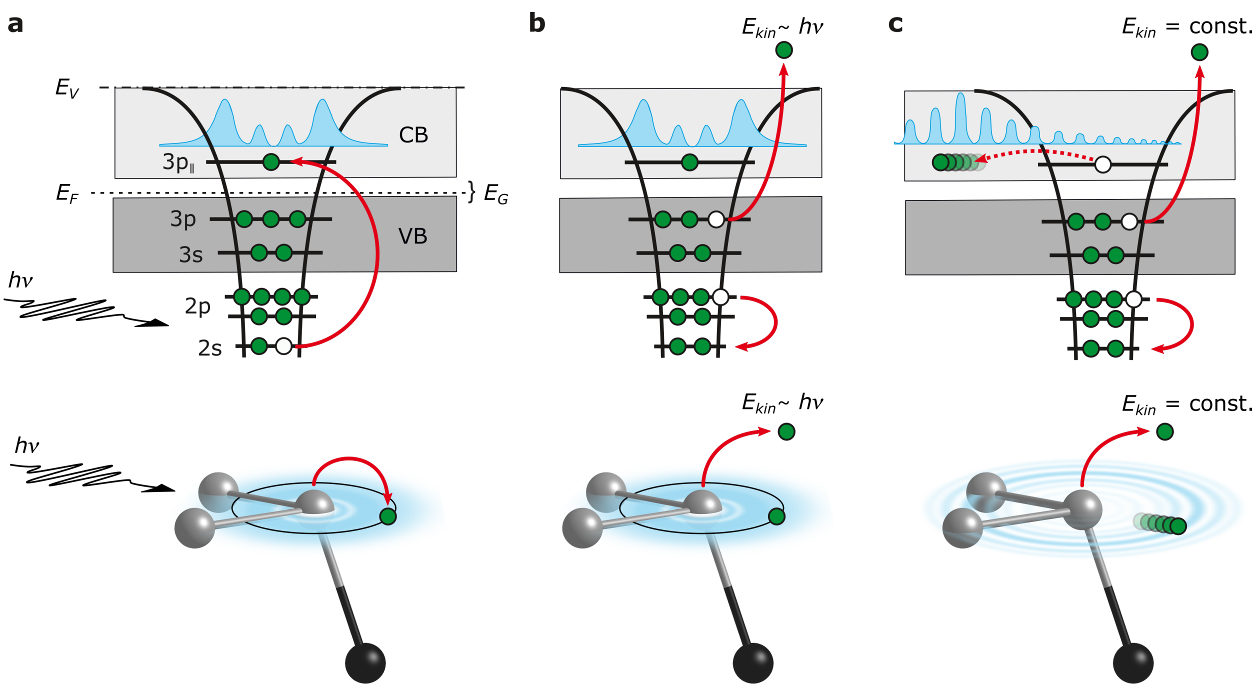

Supplementary Figure 1: Schematic illustration of the core-hole clock principle on black phosphorus, exemplarily shown for in-plane excitation by linearly polarized X-rays. Following the resonant excitation of a P 2s core electron by a photon with energy $h\nu$ into a bound, unoccupied P $3p_{\parallel}$ in-plane conduction band state (**a**), two different deexcitation channels of the subsequent autoionization decay can be observed: The Raman channel (**b**), if the resonantly excited electron remains in an atomically localized state with the kinetic energy of the ejected electron depending linearly on the photon energy and the Auger channel (**c**), if the resonantly excited electron is delocalized within the conduction band with a constant kinetic energy of the ejected electron. E_{kin} kinetic energy; E_V vacuum level; E_F Fermi level; E_G band gap energy; VB valence band; CB conduction band

LEED study of bulk BP

Using surface-sensitive low-energy electron diffraction (LEED) analysis, we determine the orientation of the single-crystalline BP samples. Before the LEED analysis, the BP samples were cleaved *in situ* at room temperature. Cleaving and LEED analysis were performed under UHV conditions at a base pressure of $\sim 2 \cdot 10^{-10}$ mbar. The measurements were performed using an OCI Vacuum Microengineering LEED and Auger electron spectrometer, model LEED 800 with an electron beam focus size of $\sim$ 1 mm. The measurements were performed with a normal incident electron beam.
Supplementary Fig. 2 shows example LEED diffraction patterns, which, based on the distances and directions between the neighboring diffraction spots, allow to distinguish the two high-symmetry in-plane crystal directions: armchair (AC) and zigzag (ZZ). The alignment of the linearly polarized X-rays with the electric field vector ($\vec{E}$) along the short AC axis ($\vec{a}_{AC}$) and along the long ZZ axis ($\vec{a}_{ZZ}$) is schematically depicted in Supplementary Fig. 2a and Supplementary Fig. 2b, respectively. To accomplish this alignment, the sample was removed from the UHV system and rotated by 90° under atmospheric pressure before being reintroduced to the UHV system and cleaved again. The measured mean reciprocal unit cell vector ratio of $\vec{a}_{ZZ}$ / $\vec{a}_{AC}$ = 0.73 is consistent with the calculated ratio $\vec{a}_{ZZ}$ / $\vec{a}_{AC}$ = 0.75 of an ideal lattice.[S1]

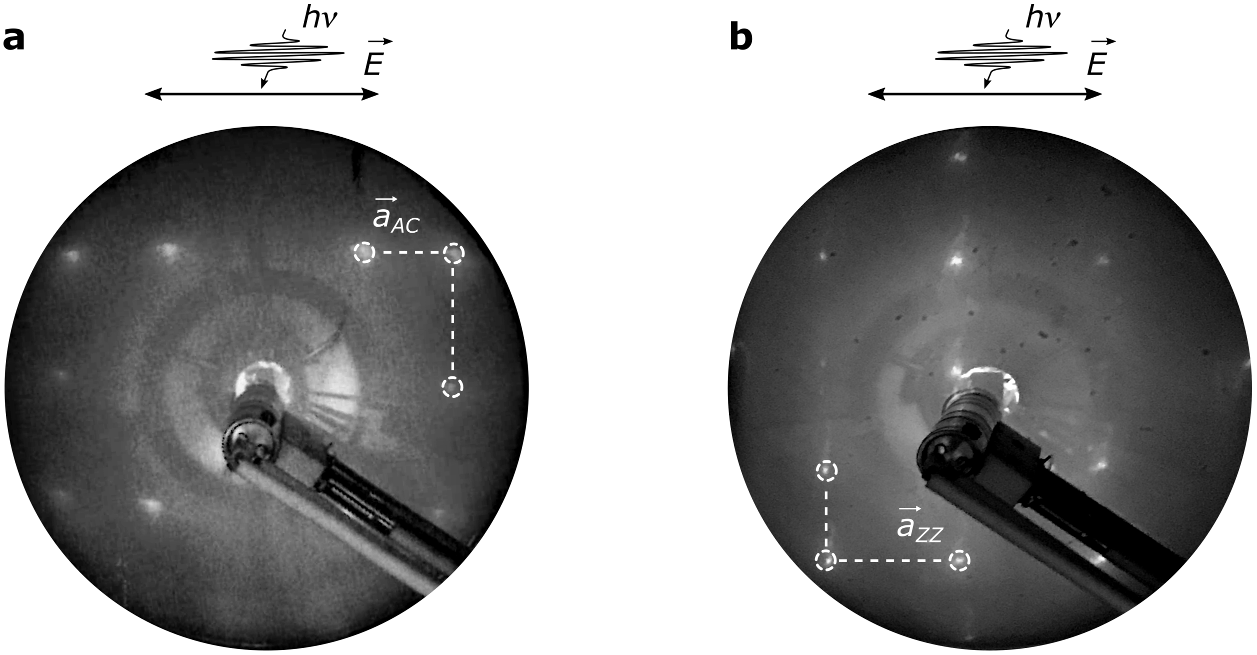

Supplementary Figure 2: LEED diffraction pattern obtained from a single crystal bulk BP surface. The reciprocal unit cell vectors $\vec{a}_{AC}$ and $\vec{a}_{ZZ}$ are depicted by dashed lines and labeled. **a** and **b** schematically show the alignment of the linearly polarized X-rays with the electric field vector ($\vec{E}$) along the armchair and the zigzag crystal direction, respectively.

Directional P $L_1L_{2,3}M_{1,2,3}$ CK autoionization spectra of BP

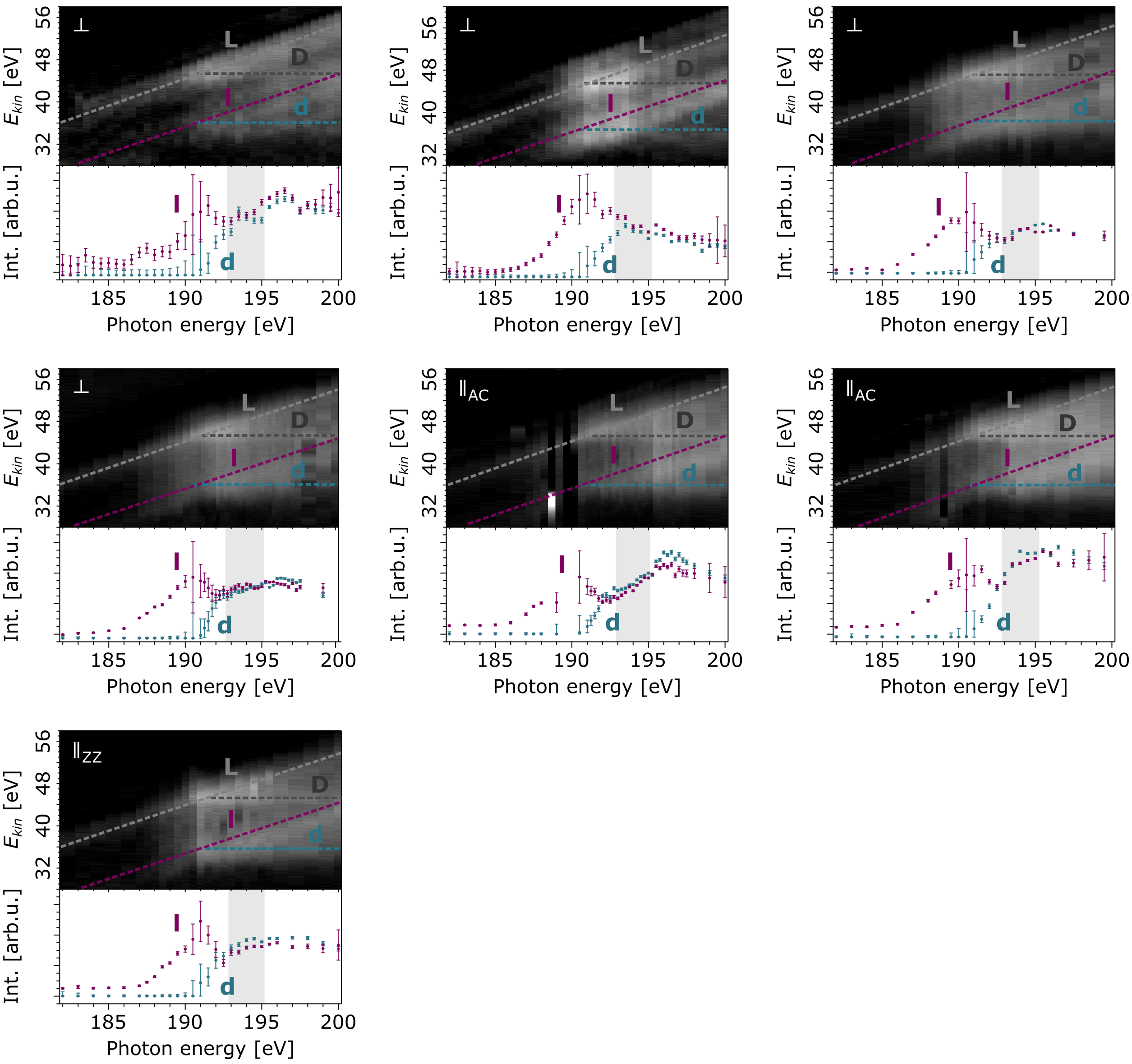

Supplementary Figure 3: Directional P $L_1L_{2,3}M_{1,2,3}$ CK autoionization spectra of BP as a function of photon energy for selective preparation of P 3p excited states in the out-of-plane direction $\perp$ as well as in the in-plane zigzag $\|_{ZZ}$ and armchair $\|_{AC}$ direction. The Raman-channels l (P $2p^{-1}3s^{-1}3p^{1}$) and L (P $2p^{-1}3p^{-1}3p^{1}$) as well as the Auger-channels d (P $2p^{-1}3s^{-1}$deloc.^{1}) and D (P $2p^{-1}3p^{-1}$deloc.^{1}) are indicated. The branching ratios of the respective l- and d-channels are plotted below each autoionization spectrum. Error bars represent the spectral fit uncertainty. The relevant photon energy region from 193 eV to 195 eV, used to extract CT times, is highlighted. The spectral contributions of the direct P 2p photoionization have been subtracted.

P L_1 and $L_{2,3}$ NEXAFS of BP

Measured near-edge X-ray absorption fine structure (NEXAFS) spectra at the P L_1- and the P $L_{2,3}$ absorption edge of the as-cleaved bulk BP sample are shown in Supplementary Fig. 4. NEXAFS spectra were measured in the total electron yield (TEY) configuration, using the photoelectron current, with the X-ray beam at normal incidence to the sample surface.
The onset of the P $L_{2,3}$ absorption edge can be seen at $\sim$ 130.2 eV as shown in Supplementary Fig. 4a. The near-edge absorption spectrum exhibits two sharp peaks in the region of 130 eV – 132 eV corresponding to excitation from the spin-orbit split P $2p_{3/2}$ and P $2p_{1/2}$ states (P 2p $\rightarrow$ 1e*)[S2].
The P L_1 absorption edge is shown in Supplementary Fig. 4b. The bold line indicates the P 2s $\rightarrow$ P 3p resonance, with the resonance maximum at 191.2 eV. An exponentially modified Gaussian peak with a FWHM of 2.64 eV was used to model the resonance shape. The relevant photon energy region from 193 eV to 195 eV used to extract the charge transfer times is highlighted.

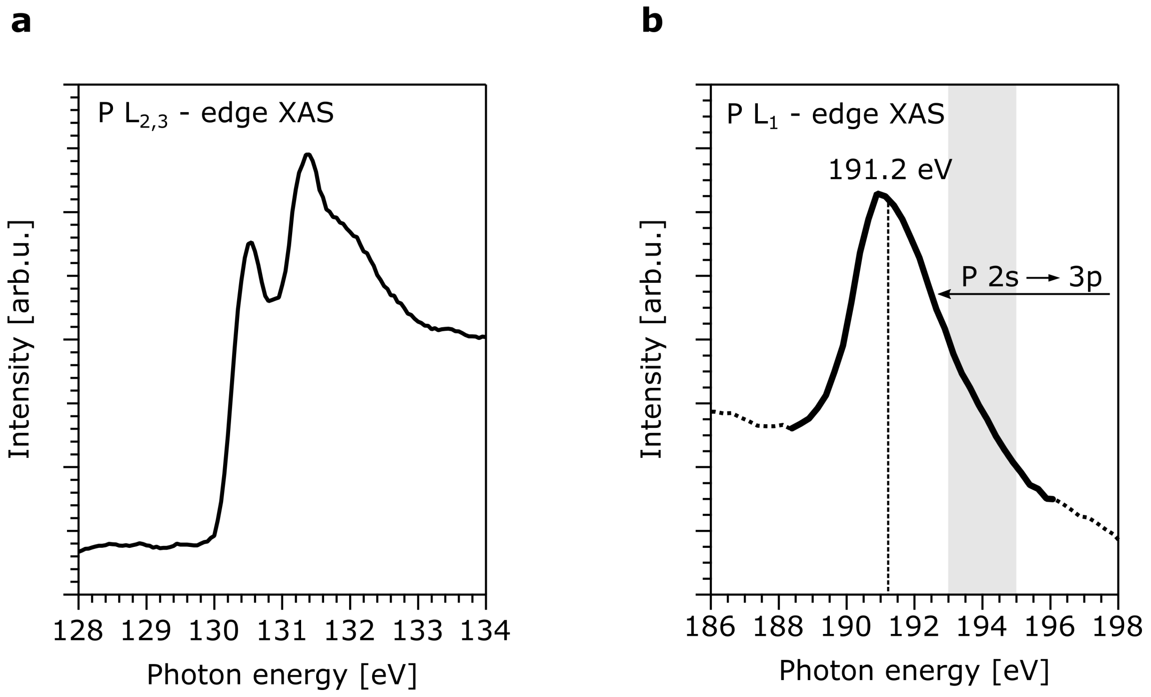

Supplementary Figure 4: Near-edge X-ray absorption fine-structure (NEXAFS) spectra of the as-cleaved BP sample at the P $L_{2,3}$ (**a**) and the P L_1 (**b**) absorption edge. In the P L_1 NEXAFS spectra, the relevant photon energy region from 193 eV to 195 eV, used to extract average charge transfer times, is highlighted.

Data analysis and spectral decomposition

All spectral intensities of the raw data were normalized to the X-ray photon flux of the incident synchrotron radiation. Below the P L_1- absorption edge, no resonant excitation occurs and the measured spectra in this photon energy (hv) range are exclusively composed of spectral features corresponding to the direct P 2p photoionization, shake-up processes and the inelastic scattering background. The background is modeled up to the direct P 2p photoionization line by a Tougaard background function:

$$F(E) = J(E) - B_1 \int_{E}^{E_{max}} J(E) \frac{E' - E}{(C + (E' - E)^2)^2} \cdot dE' \tag{1}$$

J(E) expresses the measured spectrum at energy E and *(E' - E)* expresses the electron energy loss at energy *E'*. The C parameter is kept at the standard value of 1064 eV^2. The fitting factor B_1 is adjusted to give zero intensity in a region up to 50 eV below the characteristic peak structure.[S3]
Subsequently, the determined line shape of the direct P 2p photoionization line, including the corresponding shake features, has been shifted according to the varying X-ray energy and subtracted from each spectrum. According to this procedure, for hv above the P L_1-absorption edge, only the spectral components corresponding to the P $L_1L_{2,3}M_{1,2,3}$ CK autoionization decay following the P 2s $\rightarrow$ P 3p resonant excitation remain.
The spectral decomposition is exemplary shown in Supplementary Fig. 5, for hv below the P L_1-absorption edge maximum at 175 eV and 188 eV as well as above the P L_1-absorption edge maximum at 193 eV, 194 eV and 195 eV.

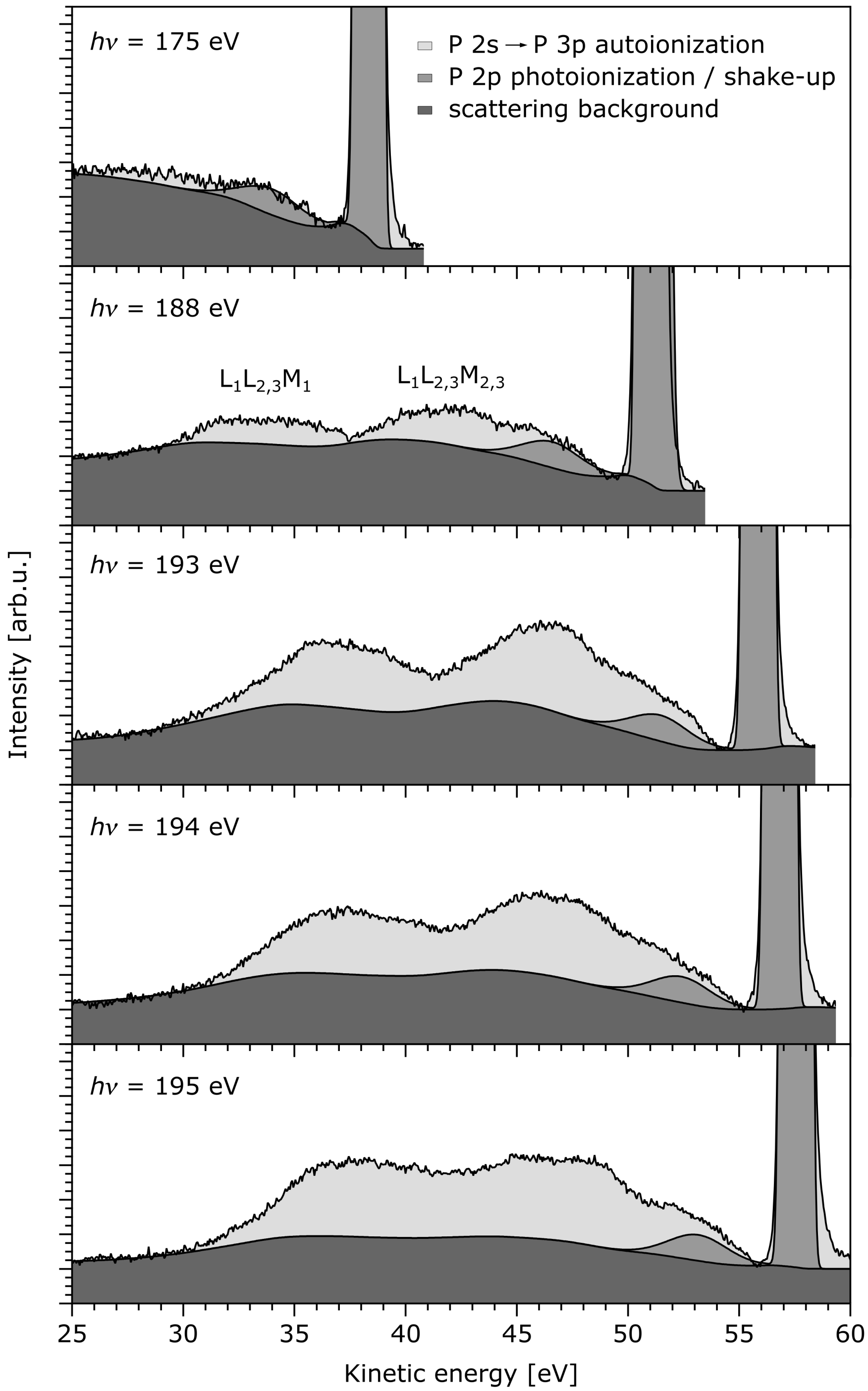

Supplementary Figure 5: Spectral decomposition and evolution of the BP autoionization spectra, exemplary shown for exciting X-ray energies (*hv*) below (175 eV and 188 eV) and above the P L_1-absorption edge maximum (193 eV, 194 eV and 195 eV). The kinetic energy region from the direct P 2p photoionization lines upwards has been subtracted.

Quantitative $L_1L_{2,3}M_{1,2,3}$ CK autoionization decay channel analysis

To quantitatively evaluate the contributions of the Raman- and the Auger channels to the autoionization spectrum, a peak fitting routine has been used. According to their origin, the charge transfer Auger channels d and D are fixed at constant kinetic energy (E_{kin}) at 36.2 ± 0.1 eV (d) and 45.2 ± 0.1 eV (D), respectively. The localized Raman channels l and L are fixed at constant binding energy (E_B) at 146.9 ± 0.1 eV (l) and 137.9 ± 0.1 eV (L). The spectral distribution of each decay channel is approximated by a Gaussian peak where the best overall fit was obtained for all four decay channel features (d, D, l, L) with a common full width at half maximum (FWHM) of 4.5 eV, while only their intensities are free parameters. The spectral shape of the final states reached, respectively the line shape of the spectral channels, reflects the convolution of the natural 2s core hole lifetime broadening, the experimental broadening and the width of the energy levels involved in the decay process, with the band-like 3s or 3p states dominating the broadening over the atomic 2p states and the 2s lifetime broadening.

Exemplary, the fitting procedure is shown in Supplementary Fig. 6 for specific photon energies below ($hv = 188$ eV) and above ($hv = 193$ eV, $hv = 194$ eV and $hv = 195$ eV) the P L_1 absorption edge and varying excitation directions. The l- and d-channel peaks are shown in purple and blue. The L- and D-channel peaks are shown in light grey and dark grey. Below the P L_1 absorption edge for out-of-plane $\perp$ (a) as well as for in-plane excitation in the zigzag direction $\|_{\mathrm{ZZ}}$ (b) and in the armchair direction $\|_{\mathrm{AC}}$ (c), only the localized Raman-channels l and L are visible.

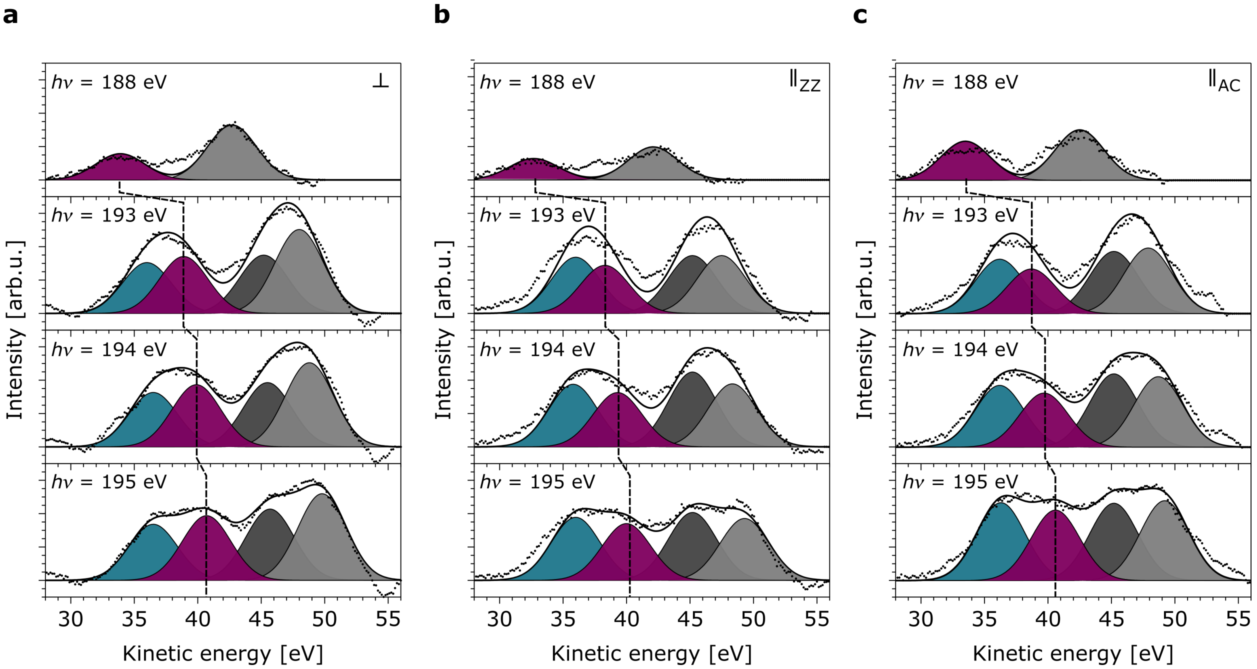

Supplementary Figure 6: Applied fitting routine to the polarization-dependent $L_1L_{2,3}M_{1,2,3}$ CK autoionization spectra of BP. Excitation into P 3p orbitals in the out-of-plane direction $\perp$ (**a**) as well as in the two in-plane directions: zigzag $\|_{ZZ}$ (**b**) and armchair $\|_{AC}$ (**c**) are shown for X-ray energies below the P L_1 absorption edge ($hv = 188$ eV) and above the P L_1 absorption edge ($hv = 193$ eV, $hv = 194$ eV and $hv = 195$ eV). Blue and purple peaks depict the d- and the l-channels respectively. Dark grey and light grey peaks depict the D- and the L-channels, respectively.

Directional CT times in bulk BP

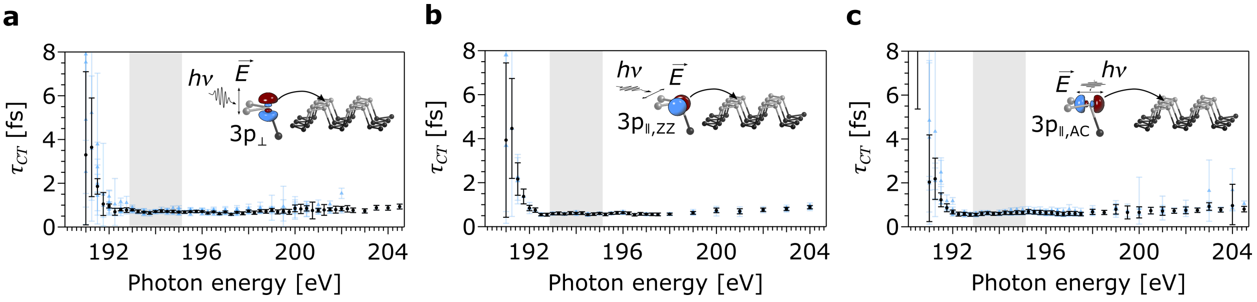

Supplementary Figure 7: Excitation photon energy dependence of CT times in BP in the out-of-plane $\tau_{\perp}$ (**a**) as well as in the in-plane zigzag $\tau_{\parallel,ZZ}$ (**b**) and the armchair $\tau_{\parallel,AC}$ (**c**) direction obtained from the related P $L_1L_{2,3}M_{1,2,3}$ spectral intensities of the l- and d-channel. Extracted τ_{CT} values from all individual CK autoionization spectra (blue triangles) were used to calculate weighted average values (black circles). Error bars for individual measurements result from Gaussian error propagation through the CHC analysis. Error bars for mean values were calculated from the standard deviation of the weighted mean. The relevant photon energy region from 193 eV to 195 eV, used to extract average charge transfer times, is highlighted.

XPS sample characterization

Before measuring the P $L_1L_{2,3}M_{1,2,3}$ CK autoionization spectra, each sample was cleaved under ultra-high vacuum ($\sim 2 \cdot 10^{-10}$ mbar) conditions and characterized by X-ray photoelectron spectroscopy (XPS) at a pressure of $2 \cdot 10^{-10}$ mbar. Supplementary Fig. 8 shows an exemplary set of XPS spectra consisting of a survey scan (a), valence band (VB) scan (b) as well as P 2p (c) and P 2s (d) core level scans. All binding energies (E_B) are referred to the Fermi level (E_F), determined from Au VB reference measurements ($E_B = 0$ eV at E_F). The survey scan is recorded at a photon energy of $hv = 800$ eV in the binding energy range from 0 eV up to 580 eV. The apparent features are attributed to the P 2p and P 2s core levels along with their respective energy loss features. The energy loss features are clearly observable up to second order and related to bulk plasmon excitation with energy of ~ 20.1 eV[S4,S5]. No traces of oxygen, nitrogen or carbon species physisorbed onto the BP surface are visible. Further, we did not detect any time-dependent spectral changes during the storage in UHV, confirming the stability of the fresh BP surface.

The VB scan allowed to estimate the VB edge $\sim 0.35 \pm 0.02$ eV below E_F by applying a linear fit in the binding energy region around 1 eV. The three distinct features visible in the binding energy range from 1 eV up to 7 eV are primarily related to 3p orbitals, with little admixture of 3s orbitals in the second feature.[S6]

The P $2p_{3/2}$ core level is found at $E_B = 129.8$ eV, well separated from the P $2p_{1/2}$ core level with a spin-orbit splitting of 0.9 eV. To model the P 2p spin-orbit split doublet, a linear background was subtracted and Gaussian peaks with identical FWHM of 0.56 eV and an intensity ratio of $1:2$ for P $2p_{1/2}$: P $2p_{3/2}$ were used. To model the P 2s core level spectrum, a linear background subtraction and a Voigt peak at $E_B = 188.5$ eV and a FWHM of 1.3 eV was used. No asymmetry in the core level peaks or signs of oxidized phosphorus species are visible in the P 2p and P 2s core level spectra,[S7] confirming the high quality of the sample and a pristine BP surface.

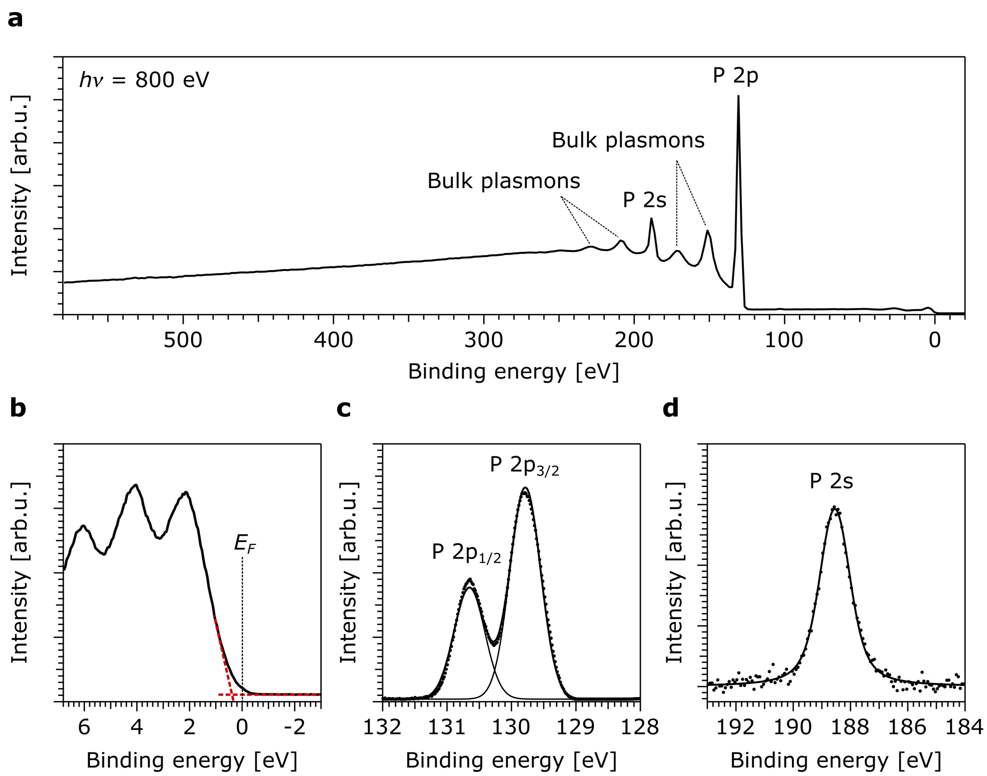

Supplementary Figure 8: Exemplary XPS characterization of one BP sample consisting of a survey scan (**a**), a BP VB scan (**b**) as well as P 2p (**c**) and P 2s (**d**) core level scans.

References

(S1) Wang, C.; Niu, D.; Liu, B.; Wang, S.; Wei, X.; Liu, Y.; Xie, H.; Gao, Y. Charge Transfer at the PTCDA/Black Phosphorus Interface. *J. Phys. Chem. C* **2017**, *121*, 18084–18094.

(S2) Jürgensen, A. The P(1s) and P(2p) XAFS Spectra of Elemental Phosphorus, Theory and Experiment. *Phys. Scr.* **2005**, *2005*, 548–551.

(S3) Tougaard, S. Practical Guide to the Use of Backgrounds in Quantitative XPS. *J. Vac. Sci. Technol. A* **2021**, *A39*, 011201.

(S4) Harada, Y.; Murano, K.; Shirotani, I.; Takahashi, T.; Maruyama, Y. Electronic Structure of Black Phosphorus Studied by X-Ray Photoelectron Spectroscopy. *Solid State Commun.* **1982**, *44*, 877–879.

(S5) David, D. G.; Godet, C.; Johansson, F. O.; Lindblad, A. Quantitative Analysis of Plasmon Excitations in Hard X-Ray Photoelectron Spectra of Bulk Black Phosphorus. *Appl. Surf. Sci.* **2020**, *505*, 144385.

(S6) Asahina, H.; Morita, A. Band Structure and Optical Properties in Black Phosphorus. *J. Phys. C: Solid State Phys.* **1984**, *17*, 1839 – 1852.

(S7) Edmonds, M. T.; Tadich, A.; Carvalho, A.; Ziletti, A.; O'Donnell, K. M.; Koenig, S. P.; Coker, D. F.; Özyilmaz, B.; Neto, A. H.; Fuhrer, M. S. Creating a Stable Oxide at the Surface of Black Phosphorus. *ACS Appl. Mater. Interfaces* **2015**, *7*, 14557–14562.
